# How Not to Do It

**Published:** 1871-02

**Authors:** 


### HOW NOT TO DO IT.

A NITROUS oxyd case is reported in the January number of the *Dental Cosmos* which is somewhat suggestive. At least the report is suggestive. A young lady calling at the Dispensary of the Philadelphia Dental College to have *just one* tooth extracted, "was advised not to have the gas administered; she insisted upon it, * * the gas was then administered.—" This reminds us of a little story: A man who had imbibed too freely, with his stomach in a state of active rebellion, was approached by a stranger who sympathizingly remarked, "My friend, there seems to be a load on your stomach. I have been relieved, in such a case, by running my finger down my throat." "Look here," was the reply, "is this *my* puke or *your'n?*" Now is the *desire* of a patient, even though she be a young lady "aged 21 years," to overrule the *opinion* of a whole college? And is it likely that young operators will be taught to make their enlightened judgments the rule of action, regardless of the wishes of patients, by such an example?

The patient "became asphyxiated." And now what? Marshall Hall's ready method of artificial respiration? Not exactly.

"Insufflation." That is, the "demonstrator forced the air into her lungs a number of times by applying his mouth to hers." The young mother who saved her aged father from starvation, in the tyrant's prison, by the lacteal fluid secreted from her own blood, can not be too enthusiastically commended. The loafer who found the wounded man dying of thirst, on the lake shore, yet only offered him an aqueous excretion of his own body, would be held in everlasting execration, only that we can't believe the story. But if true, he was not a whit behind the "demonstrator," either in sense or cleanliness. Think of dosing a poor, unconscious girl with a filthy mixture so deadly that it will instantly extinguish the flame of a candle, or even of hydrogen! It will be orthodox for young practitioners hereafter to apply their mouths to the mouths of patients, if they are good looking ladies not over "21 years of age;" for that is the way they have been taught at college.

We are told "that the gas was perfectly pure." Analyzed by the professor of chemistry? No! given to other patients to see if it would kill them. Let toxicologists make a note of this new mode of analysis. Further, it is said, "The administration was properly conducted." Well,—may be! "The untoward results in the case were due to hypertrophy of the heart, inherited from the patient's mother, and aggravated by the excessive use of coffee." It was Mrs. Partington, who, in speaking of hypertrophy of the cuticle, said: "Some things is Herod Dittery and some isn't. Now my corns is inherited from my good man's stepmother, and aggravated by the excessive wearin' of tight shoes."

